# Dr. Massey’s Case of Radical Cure of Puerperal Ascites, by Spontaneous Exudation through the Parietes of the Abdomen

**Published:** 1842-05-21

**Authors:** J. Massey

**Affiliations:** Surgeon, Nottingham


### ANALECTA.

***Radical cure of Puerperal Ascites, by Spontaneous Exudation through the Parieties of the Abdomen.*** By J. Massey, Esq., Surgeon, Nottingham.—Nov. 17, 1841. Mrs. Lee, living in Nottingham, is thirty-nine years old, and the mother of three children ; she is of a sallow complexion, but has always had good health; previous to her accouchement, which took place three months ago, she was troubled with cough, palpitation, dyspnœa, headach, scanty secretion of urine, with œdematous swellings of the lower limbs, and considerably more abdominal enlargement than on former occasions. Immediately after delivery the abdomen became enormously distended, and it was with much difficulty that she could move about in bed. She was admitted a patient under me at the General Dispensary ; four days after her confinement she was visited by Dr. Taylor, the resident surgeon to that institution, who prescribed for her diuretic and cathartic medicines: I visited the case subsequently. She continued these remedies for about two months, which increased the secretion of urine and evacuated the bowels ; still the tension and enlargement of the abdomen continued undiminished ; paracentesis was considered advisable, which was delayed as a last resource. A few days afterwards, without any assignable cause, she began (to use her own phrase) to "sweat" so much about the abdomen during the night that she could wring the wet off her flannel petticoat: this was the case every night ; and in order more effectually to keep herself dry, she wrapped round her body an additional quantity of flannel, but on the following morning they were invariably completely saturated with the moisture; no perspiration was in the least manifested in other parts of the body ; the secretion of urine was not scanty, being little more than the usual quantity when in health. The purgative and diuretic medicines were continued. I very much regret that no analysis was ever made of the fluid.

25. It is now about a month since she perceived first the exudations from the abdomen ; they have regularly continued, and there is a very considerable reduction in her circumference; a month ago her stays could not be laced, nor would they meet by twelve to fourteen inches; now they meet very easily ; the secretion of urine continues free as before, and the bowels are open.

Dec. 4. Continues improving. Continue medicines.

14. She states that there has been much less "sweating about the bowels" the last two or three nights, and she has in consequence increased in size.

Jan. 14, 1842. She is now quite well ; suckles her child, which is healthy, and follows her usual occupation as charwoman. I have briefly reported the above case, which is important: I make no further comments upon it, but leave it for the consideration of your numerous readers, to whom I shall be glad to communicate any further particulars, should they wish it, as the person still continues well, and at her usual occupation in this town.—*Lon. Lancet*, March 5.

